# Exploring Psychosocial Dynamics Underpinning Driver Identity in an Older Adult Sample

**DOI:** 10.3390/geriatrics7060122

**Published:** 2022-10-25

**Authors:** Andrew K. Lee, Theresa L. Scott

**Affiliations:** School of Psychology, The University of Queensland, St Lucia, QLD 4072, Australia

**Keywords:** older adults, driving, identity, driving cessation, mobility, transport

## Abstract

Many older adults consider driving a crucial aspect of their daily routine and the prospect of driving cessation to be disruptive to their current lifestyle. Driving cessation is associated with multiple adverse consequences, including poorer health trajectories, and increased depressive symptoms. Research suggests that driving cessation may be disruptive to identity. This study aimed to explore the characteristics that are associated with driver identity and whether identity impacted people’s readiness for mobility changes. Of interest was whether stopping driving was perceived as either a positive or negative event. Participants, (N = 410) older adults recruited via Prolific survey panel between July and November 2021, responded to questions about transport and travel behaviors, driver identity, and perceptions of mobility changes. Driving cessation was generally perceived as a negative change. However, individuals with self-reported low readiness for mobility change also had higher overall scores for Identity, and for the subscales, Centrality and Ingroup Affect. These findings suggest that people with more concerns for mobility transition may think about and have more of an emotional investment regarding driving. The findings provide novel insight into the psychosocial dynamics of driving and the factors that influence driver identity, however further research, co-designed with older drivers and retired drivers is required.

## 1. Introduction

Many older adults consider driving a crucial aspect of their daily routine and the prospect of driving cessation to be disruptive to their current lifestyle [1,2]. The perceived importance of driving is understandable as family, friends, work, and services are often spread out over long distances, and personal vehicles offer a convenient way to reach these destinations [3,4]. Consequently, access to services and social activities may be contingent upon driving for many people [5]. Aside from satisfying the practical needs of mobility, driving also influences a person’s sense of self [6,7,8,9]. We aimed to better understand the factors that underpin the psychosocial relationship between an individual and driving and how these may influence their perception of driving cessation. Understanding such factors may help with developing individualized plans to support people who must transition to driving retirement.

Declining physical health and cognition are primary reasons for driving cessation in older adults [10]. In addition, people who drive less routinely are also more likely to stop driving sooner later in life. That is, people who regulate their driving or have alternative drivers or more transport options may be more likely to cease driving than those who are more prolific car users [11]. However, compared to their counterparts living in metropolitan areas, older adults living in remote areas may have fewer options for alternative transport.

Driving cessation may be an inevitable, although unexpected, part of ageing. Men and women can expect to live seven to ten years, respectively, beyond their ability to drive safely [12]. Even when accounting for age and health status, driving cessation was predictive of poorer health trajectories and institutionalization [13]. Moreover, some former drivers experienced social isolation and increased depressive symptoms [14,15]. Fonda and colleagues [14] also noted that some individuals who had ceased driving, but had a partner still capable of driving, experienced depressive symptoms.

Interestingly, Musselwhite and Shergold [16] found that planning for driving retirement often mitigated the adverse effects of driving cessation. Older women were more open to mobility change and sought information on alternative transport at an earlier time in their lives than older men. Furthermore, older women saw alternative transport as an opportunity to learn new skills, while older men equated it to a loss of status [16,17]. As a result, older women generally seemed to have adapted well and maintained their quality of life after driving cessation [16]. By contrast, the process of driving cessation was more abrupt in older men, who often reported a negative change to their quality of life after driving cessation [16]. 

Previously, Musselwhite and Haddad [8] noted that older men tended to focus on the emotional aspects of driving and how it enhanced their social standing and gave them a sense of commonality with others. Meanwhile, women tended to focus on the practical aspects of driving and how it allowed them to fulfil their family obligations as a parent and caregiver [8]. Indeed, driving is often associated with cultural expectations of masculinity and the need to demonstrate to others that one is in control and accomplished in life [18,19]. Perhaps over time this belief may have developed into a form of emotional attachment to the car and the driving role. 

Tajfel and Turner’s social identity theory is a useful framework for understanding how the relationship between driving and identity is developed. Social identity theory proposes that a person’s identity is formed within the context of their group membership(s) [20]. Groups are a source of pride and offer a sense of closeness with others and may include characteristics that are determined at birth (e.g., sex and ethnicity) [20]. Individuals may also choose their group membership (e.g., scientist or tennis player) and generally gravitate towards groups valued by others [20]. 

Cameron [21] further suggests that three distinct factors influence the strength of attachment to a group. Centrality refers to the salience of group membership in a person’s consciousness [22,23]. Ingroup affect relates to the emotional valence that is derived from group membership. Ingroup ties refers to the perceived closeness and similarity one shares with other group members. 

Driving has been seen as a symbol of youthfulness, competency, masculinity, and independence by many older adults [8,24,25,26,27]. Conversely, driving cessation is seen to imply the onset of old age and dependency, characteristics that are often stigmatized in society [9,28,29]. Accordingly, driving is likely to be an integral part of identity for many older adults as it represents to others that one is still a healthy and capable member of society [9]. By contrast, driving cessation is often met with resistance as it marks a permanent shift to a less desirable group and may also imply exclusion from one’s community [9]. Even so, there seem to be differences in how people cope with driving cessation. For example, having resilience and being able to adapt to challenging situations may be an important factor in how people respond to driving cessation. By evaluating the psychosocial dynamics of driving it will be possible to determine how “driver” identity is formed and influenced. More importantly, contingency plans could be personalized for those with greater attachment to the car and therefore most at risk of experiencing adverse outcomes following driving cessation. 

Existing literature has focused primarily on the implications of driving cessation in older adults. The research has shown that stopping driving is associated with numerous adverse consequences including, poorer health trajectories, institutionalization, social isolation and increased depressive symptoms [13,14,15]. Some researchers have suggested that the loss of identity may explain these adverse outcomes [8,9]. Indeed, some studies have demonstrated that older adults considered driving cessation as a life transition that would be impactful to their self-image and daily routine [2,5,9]. Research is required to determine the factors that underpin the development of driver identity to support individuals in the transition from driver to non-driver, if driving cessation is indicated. Further, knowing the contributing factors may provide an indication of the degree to which ceasing driving would change areas of an individual’s life, and to encourage engagement in planning for driving retirement to lessen the distress often experienced by driving cessation. To the best of the authors’ knowledge, little research has been conducted on the factors that influence the formation of driver identity and more importantly the factors that impact whether stopping driving is perceived as a negative or positive event. To address this gap in knowledge we aimed to answer the following research questions:

- Do driving characteristics (history, roles: principal or non-principal driver, frequency, perception of driving standard, availability of alternative transport), driver identity, perception of driving cessation and resilience differ across gender, residential area, principal and non-principal drivers, and individuals with different degrees of readiness for mobility transition?
- Which characteristics are associated with driver identity and does identity impact people’s readiness for mobility change and their perception of driving cessation as either a positive or negative event?

## 2. Methods

### 2.1. Participants 

The study was a cross-sectional survey-based assessment. Participants were recruited via the Prolific survey panel between July 2021 and November 2021. To be eligible, participants had to be a minimum of 60 years of age and a current driver. Participants accessed a link through the Prolific website which contained an information sheet, consent form and survey hosted on Qualtrics. They were reimbursed $5.07 USD for their time. An online survey was completed by 410 individuals residing in the United States, aged 60–83 years whose driving history spanned 20.00–68.00 years. The sample size was adequate in accordance with recommendations specified by [30], *N* > 50 + 8 m where m is the number of variables under investigation. The study had institutional ethics approval from The University of Queensland, Australia. 

### 2.2. Materials

The online survey comprised of demographic questions as illustrated in Table 1, and questions about respondents’ transport and travel behaviors, driving identity, and driving cessation, as outlined below. The survey was administered via Qualtrics© online survey system. 

### 2.3. Short Answer Responses

To determine availability of alternative transport, we asked participants to elaborate on the following dichotomized question, “Are there other modes of transportation easily accessible or practical for you to use instead of driving?”

#### 2.3.1. Readiness for Mobility Transition

The Assessment for Readiness for Mobility Transition [31] is a 24-item scale used to determine the degree to which one is attitudinally ready for mobility transition. The ARMT- Total Score (ARMT-TS) has demonstrated good internal consistency and split half reliability (each α = 0.88). An example item was as follows, *“It is devasting for older people to have someone take away their car keys”.* Items were measured using responses of 1 (strongly disagree) to 5 (strongly agree). The items were collapsed and used to determine a mean score of readiness. A cut-off score of 3.57 was recommended by Meuser and Colleagues as it represented one deviation above the validation sample mean during their initial investigation. Higher scores indicated less openness for mobility change. Individuals with ARMT-TS > 3.57 were considered to be high risk and said to have a low readiness for mobility change. Individuals with ARMT-TS between 2.29 to 3.57 were considered to be average risk and said to have mixed readiness for mobility change. Individuals with ARMT-TS < 2.29 were considered to be at low risk and said to have high readiness for mobility change. People with lower readiness (higher scores) were less likely to be prepared for driving retirement and were more likely to report feeling threatened by the prospect of driving cessation. Meanwhile, people with lower scores (higher readiness) were more open to mobility change and less concerned by the prospect of driving cessation. People with mixed readiness have reservations about mobility change but are more open to changes to their lifestyle when to compared to individuals with low readiness.

#### 2.3.2. Driver Identity

Driving identity was measured via a modification of The Three-Factor Model of Social Identity scale [21]. This scale is a 12-item measure was used to assess the strength of identity through three subscales: centrality, ingroup affect and ingroup ties, and a total score overall. Each of the subscales (centrality, α = 0. 75, ingroup affect, α = 0. 70, ingroup ties, α = 0. 78, and total score (α = 0. 83) have demonstrated adequate to good internal consistency [32]. Each subscale had 4 items which allowed for the insertion of a variable name. 2 items were reversed scored. An example item on the centrality subscale was as follows, *“I often think about being a driver”.* A reverse scored item on the centrality subscale was as follows, *“The fact I am a driver rarely enters my mind”..* An example item on the ingroup affect subscale was as follows, *“Generally I feel good about myself when I think about being a driver”.* A reverse scored item on the ingroup affect subscale was as follows, *“I don’t feel good about being a driver”.* An example item on the ingroup ties subscale was as follows, *“I have a lot in common with other drivers”.*. A reverse scored item on the ingroup affect subscale was as follows, *“I don’t feel a strong sense of being connected to other drivers”.* Items were measured using responses of 1 (strongly disagree) to 7 (strongly agree). Higher scores on each of the scale indicated greater identity. The items were collapsed and used to determine a mean score of identity (total score).

### 2.4. Single-Item Measures

Several single-item measures were developed for this study to measure individual’s self-reported driving behaviors. “To assess how principal drivers viewed driving, the following item was used, “I *do not mind doing the majority of driving in my household”* using responses of 1 (strongly disagree) to 5 (strongly agree). To assess how people viewed their standard of driving and of health the following item(s) were used. *“How would you describe your (driving standard) (health) compared to other people of the same age and gender?”* Driving standard and perceived health were measured using responses of 1 (much worse) to 5 (much better). Higher scores indicated better driving standard and perceived health. To assess how people perceived driving cessation, the following item was used, *“Overall, do you perceive driving cessation as a negative or positive change?”* Participants rated driving cessation with responses of 0 (extremely negative) to 10 (extremely positive). Higher scores indicated more positive endorsement of driving cessation.

#### 2.4.1. Trait Assessment of Resilience

Resilience was measured using The Trait Assessment of Resilience [33]. Resilience was measured to determine whether this trait had any influence on the psychosocial dynamics of driving. The scale consisted of 7 item scale, with 3 reversed scored items. The scale has demonstrated sound convergent and concurrent validity with previous measures [33]. An example item on the scale, *“Generally, when others may give up, I stand strong and keep fighting.* “A reverse scored item was as follows, “*I don’t believe I have the emotional strength to cope with life stress.*” Items were measured using responses of 1 (disagree) to 4 (strongly agree). Higher scores indicated greater resilience. The items were collapsed and used to determine a mean score of trait resilience.

#### 2.4.2. Data Analysis Strategy

No variables were missing more than 7% of their data so available-cases analysis was used for this study [34]. Differences between categorical variables were assessed using chi-square tests of independence. All expected cell frequencies were greater than five. Mahalanobis distance was used to assess for multivariate outliers. Normality was assessed by inspection of Q-Q plots. Welch corrections were applied when homogeneity of variance was violated. Multiple comparisons were adjusted using holm-Bonferroni sequential corrections [35]. ** Data were transformed initially to account for outliers and normality violations but did not help with violation and did not affect the interpretation of our results so untransformed data were presented in results. Spearman’s correlation robust to outliers was used to assess relationships of ordinal variables (our single item measures).

## 3. Results

### 3.1. Driving Characteristics (History)

**Figure 1:**
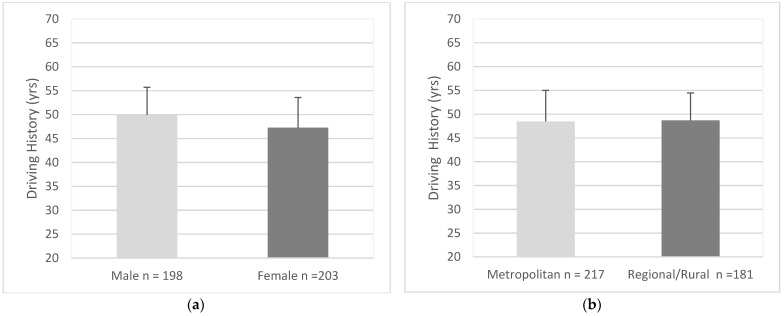

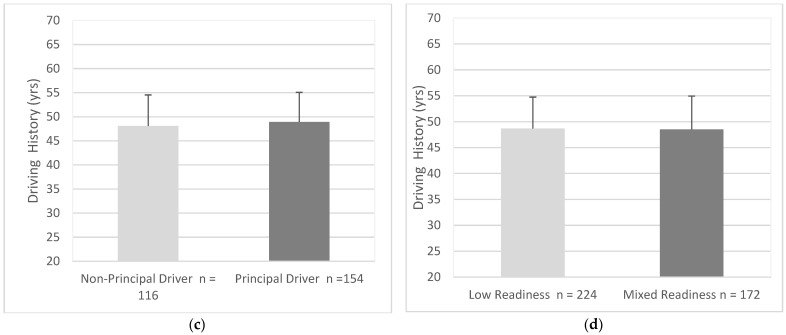
Differences in driving history between groups; (**a**) Gender, (**b**) Residential Area, (**c**) Driving Roles, (**d**) Readiness. Error bars represent standard deviation.

In terms of driving history, males (*M* = 49.95 years, *SD* = 5.75 years) had significantly more driving experience than females (*M* = 47.27 years, *SD* = 6.30 years), (95% CI, 1.50 to 3.87), *t* (399) = 4.45, *p* < 0.001. (Figure 1a). Drivers from metropolitan areas (*M* = 48.46 years, *SD* = 6.54) and regional/rural areas had a similar level of driving experience (*M* = 48.71 years, *SD* = 5.76 years), (95% CI, −1.47 to 0.98), *t* (396) = 0.40, *p* = 0.688 (Figure 1b). Principal drivers (*M* = 48.08 years, *SD* = 6.45 years) and non-principal drivers had a similar level of driving experience (*M* = 48.91 years, *SD* = 6.15 years), (95% CI, −2.35 to 0.69), *t* (268) = 1.07, *p* = 0.283 (Figure 1c). Drivers with low readiness (*M* = 48.70 years, *SD* = 6.04) and drivers with mixed readiness had a similar level of driving experience (*M* = 48.52 years, *SD* = 6.41), (95% CI, −1.06 to 1.41), *t* (394) = 0.28, *p* = 0.783 (Figure 1d).

### 3.2. Driving Characteristics (Roles)

**Figure 2:**
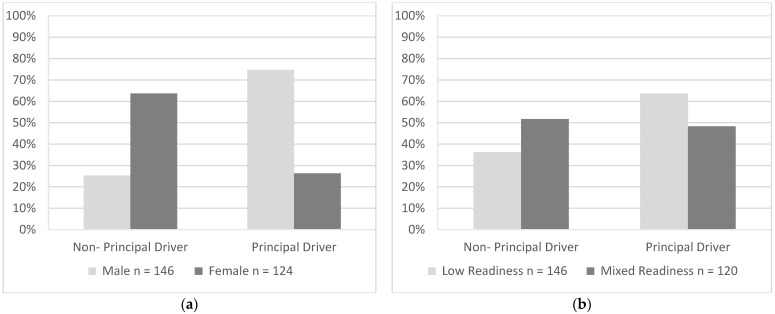
Driving Roles as endorsed by participants in each group that use driving as their primary mode of transport and had other drivers in their household. Percentages represented proportions of participants in each group who responded “yes” or “no” to the following item, “Do you do the majority of driving in your household”. (**a**) Gender, (**b**) Readiness.

For this sample of drivers, a significantly higher proportion of males (75%) reported to be principal drivers in the household compared with females (36%), χ^2^(1, *N* = 270) = 40.28, *p* < 0.001 (Figure 2a). A significantly higher proportion of drivers with low readiness were principal drivers (64%) when compared with drivers with mixed readiness (48%), χ^2^(1, *N* = 266) = 6.34, *p* = 0.012 (Figure 2b).

### 3.3. Driving Characteristics (Frequency)

**Figure 3:**
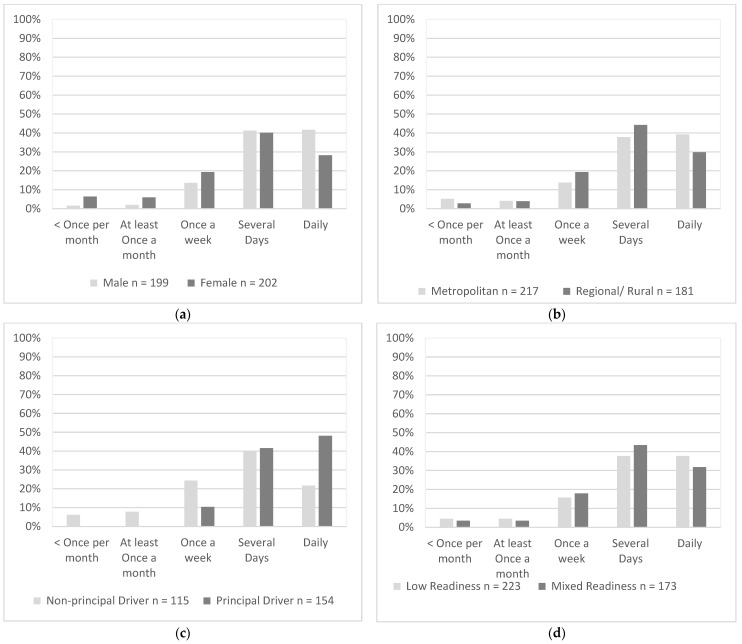
Driving Frequency as endorsed by participants in each group; (**a**) Gender, (**b**) Residential Area, (**c**) Driving Roles, (**d**) Readiness. Percentages represented proportions of participants in each group who endorsed that option.

A significantly higher proportion of males (42%) drove daily when compared with females (28%), χ^2^(1, *N* = 401) = 17.25, *p* = 0.002 (Figure 3a). There was a similar proportion of drivers from metropolitan areas (39%) and remote areas (30%) who drove daily, χ^2^(1, *N* = 398) = 6.62, *p* = 0.157 (Figure 3b). A significantly higher proportion of principal drivers (48%) drove daily compared to non-principal drivers (20%), χ^2^(4, *N* = 269) = 41.69, *p* < 0.001. (Figure 3c) There was a similar proportion of daily drivers with low readiness (38%) and mixed readiness (32%), χ^2^(1, *N* = 396) = 2.53, *p* = 0.639 (Figure 3d).

### 3.4. Driving Characteristics (Perceived Driving Standard)

**Figure 4:**
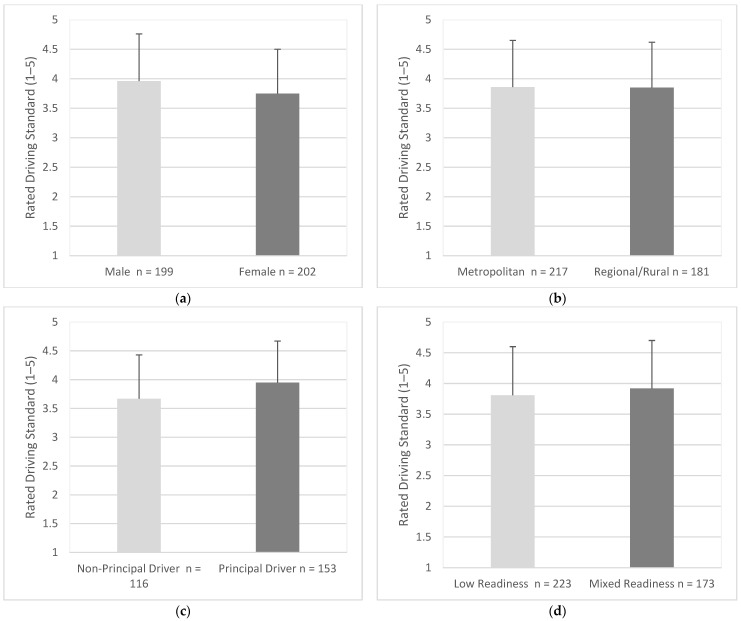
Perceived driving standard when compared to individuals of the same age and gender as endorsed by participants in each group; (**a**) Gender, (**b**) Residential Area, (**c**) Driving Roles, (**d**) Readiness. Responses were measured using responses of 1 (much worse) to 5 (much better). Error bars represent standard deviation.

Males (*M* = 3.96, *SD* = 0.80) were significantly more likely to say they were ‘better’ drivers when compared to individuals of the same age and gender than females (*M* = 3.75, *SD* = 0.75), (95% CI, 0.07 to 0.37), *t* (399) = 2.81, *p* = 0.005 (Figure 4a). Drivers in metropolitan areas (*M* = 3.86, *SD* = 0.79) and drivers in regional/rural areas (*M* = 3.85, *SD* = 0.77) were similar in how they perceived their driving standard (95% CI, −0.15 to 0.16), *t* (396) = 0.08, *p* = 0.936 (Figure 4b). Principal drivers (*M* = 3.95, *SD* = 0.72), were significantly more likely to say they were better drivers than non-principal drivers (*M* = 3.67, *SD* = 0.77), (95% CI, −0.46 to −0.10), *t* (267) = 3.01, *p* = 003 (Figure 4c). Drivers with low readiness (*M* = 3.81, *SD* = 0.79) and mixed readiness were similar in how they perceived their driving standard (*M* = 3.92, *SD* = 0.78), (95% CI, −0.27 to 0.04), *t* (394) = 1.41, *p* = 0.159 (Figure 4d).

### 3.5. Driving Characteristics (Availability of Alternative Transport)

**Figure 5:**
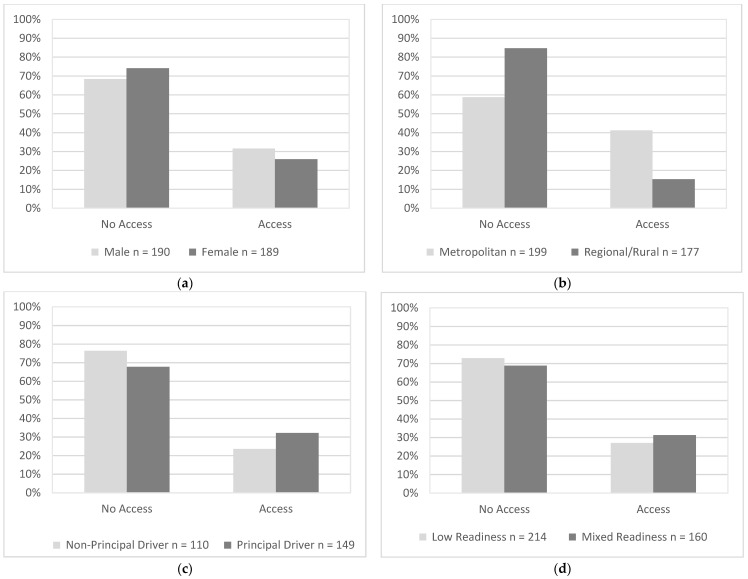
Availability of alternative transport as endorsed by participants in each group that use driving as their primary mode of transport. Percentages represent proportions of participants in each group who responded “yes” or “no” to the following item, “*Are there other modes of transportation easily accessible or practical for you to use instead of driving?—If answered driving was their primary mode of transport.*” (**a**) Gender, (**b**) Residential Area, (**c**) Driving Roles, (**d**) Readiness.

The availability of alternative transport was similar for males (32%) and females (26%), χ2(1, N = 379) = 1.48, *p* = 0.224 (Figure 5a). Drivers in metropolitan areas (42%) had significantly greater availability of alternative transport than drivers in regional/regional communities (15%), χ2(1, N = 376) = 30.65, *p* < 0.001 (Figure 5b). Availability of alternative transport was similar for principal drivers (32%) and non-principal drivers (24%), χ2(1, N = 259) = 2.28, *p* = 0.131 (Figure 5c), and for drivers with low readiness scores (27%) and mixed readiness scores (31%), χ2(1, N = 374) = 0.77, *p* = 0.381 (Figure 5d).

### 3.6. Readiness for Mobility Transition

**Figure 6:**
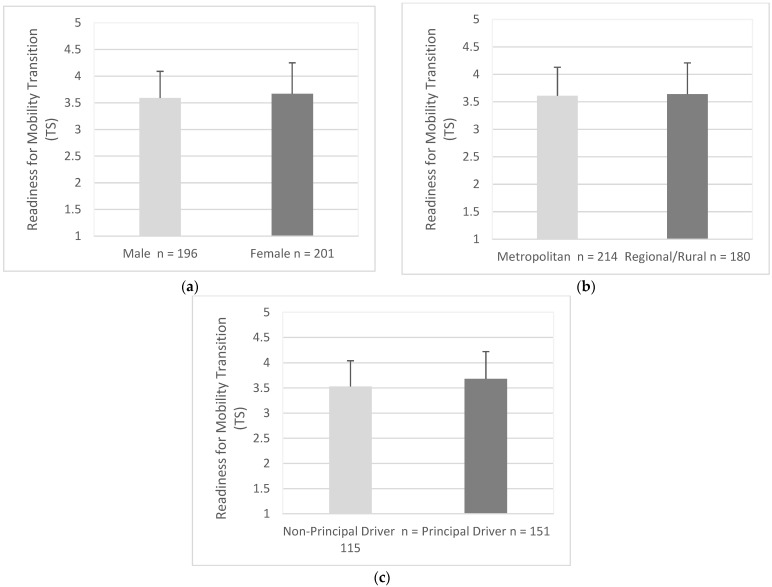
Mean score for readiness as endorsed by participants in each group; (**a**) Gender, (**b**) Residential Area, (**c**) Driving Roles. Items were measured using responses of 1 (strongly disagree) to 5 (strongly agree). Error bars represent standard deviation.

Males (M = 3.59, SD = 0.50) had a similar level of readiness for mobility transition to females (M = 3.67, SD = 0.58), (95% CI, −0.19 to 0.02), t (395) = 1.52, *p* = 0.128 (Figure 6a). Drivers from metropolitan areas (M = 3.61, SD = 0.52) had a similar level of readiness to drivers in regional/rural areas (M = 3.64, SD = 0.57), (95% CI, −0.14 to 0.08), t (392) = 0.54, *p* = 0.587 (Figure 6b). Principal drivers (M = 3.68, SD = 0.54) had a lower level of readiness when compared to non-principal drivers (M = 3.53, SD = 051.), (95% CI, −0.28 to −0.02), t (264) = 2.33, *p* = 0.021 (Figure 6c).

### 3.7. Driving and Identity factors

**Figure 7:**
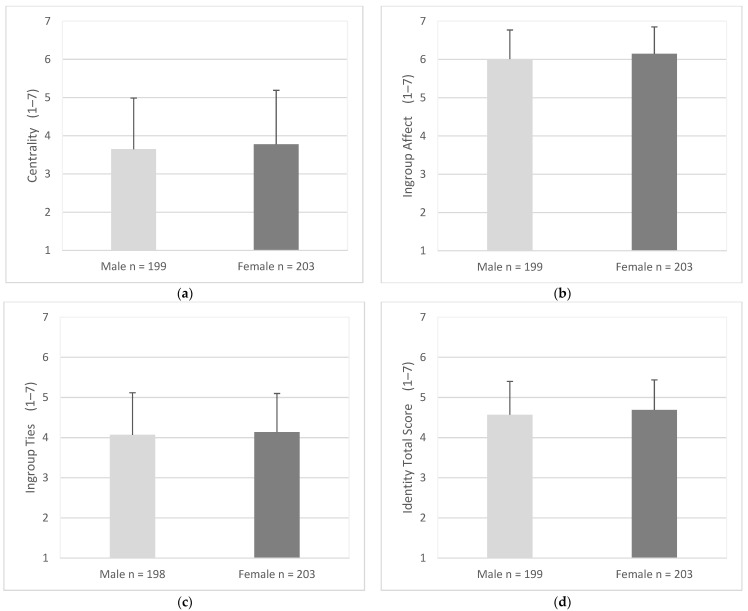
Mean score for identity factors and total scores as endorsed by male and female participants. Items were measured using responses of 1 (strongly disagree) to 7 (strongly agree). Error bars represent standard deviation. (**a**) Centrality, (**b**) Ingroup Affect, (**c**) Ingroup Ties, (**d**) Identity Total Score.

Scores of Centrality of driving identity were similar for males (M = 3.65, SD = 1.34) and females (M = 3.79, SD = 1.41), (95% CI, −0. 40 to 0.14), t (400) = 0.94, *p* = 0.348 (Figure 7a). Ingroup affect scores were similar for males (M = 6.01, SD = 0.76) and females (M = 6.15, SD = 0.70), (95% CI, −0.28 to 0.00), t (400) = 1.94, *p* = 0.108 (Figure 7b). Ingroup ties scores were similar for males (M = 4.07, SD = 1.05) and females (M = 4.14, SD = 0.96), (95% CI, −0.27 to 0.13), t (399) = 0.72, *p* = 0.348 (Figure 7c). Total scores for Identity were similar for males (M = 4.57, SD = 0.83) and females (M = 4.69, SD = 0.75), (95% CI, −0. 27 to 0.04), t (400) = 1.47, *p* = 0.216 (Figure 7d).

**Figure 8:**
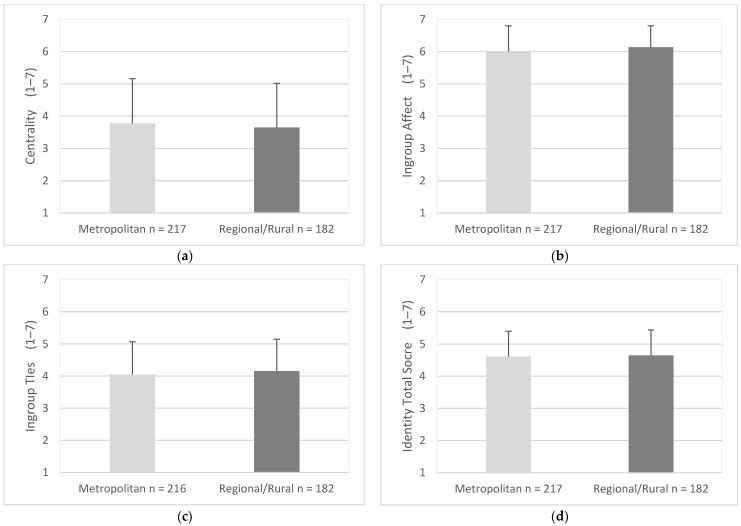
Mean score for identity factors and total scores as endorsed by drivers from metropolitan and regional/rural areas. Items were measured using responses of 1 (strongly disagree) to 7 (strongly agree). Error bars represent standard deviation. (**a**) Centrality, (**b**) Ingroup Affect, (**c**) Ingroup Ties, (**d**) Identity Total Score.

Scores of centrality were similar for drivers in metropolitan areas (M = 3.65, SD = 1.34) and drivers in regional/rural areas (M = 3.78, SD = 1.38), (95% CI, −0.15 to 0.40), t (397) = 0.90, *p* = 0.372 (Figure 8a). Ingroup affect scores were similar for drivers in metropolitan areas (M = 6.01, SD = 0.79) and drivers in regional/rural areas (M = 6.14, SD = 0.66), (95% CI, −0.28 to 0.01), t (397) = 1.80, *p* = 0.072 (Figure 8b). Ingroup ties scores were similar for drivers in metropolitan areas (M = 4.05, SD = 1.02) and drivers in regional/rural areas (M = 4.16, SD = 0.99), (95% CI, −0.31 to 0.09), t (396) = 1.06, *p* = 0.292 (Figure 8c). Total scores for Identity were similar for drivers in metropolitan areas (M = 4.61, SD = 0.79) and drivers in regional/rural areas (M = 4.61, SD = 0.79), (95% CI, −0.20 to 0.12), t (397) = 0.50, *p* = 0.619 (Figure 8d).

**Figure 9:**
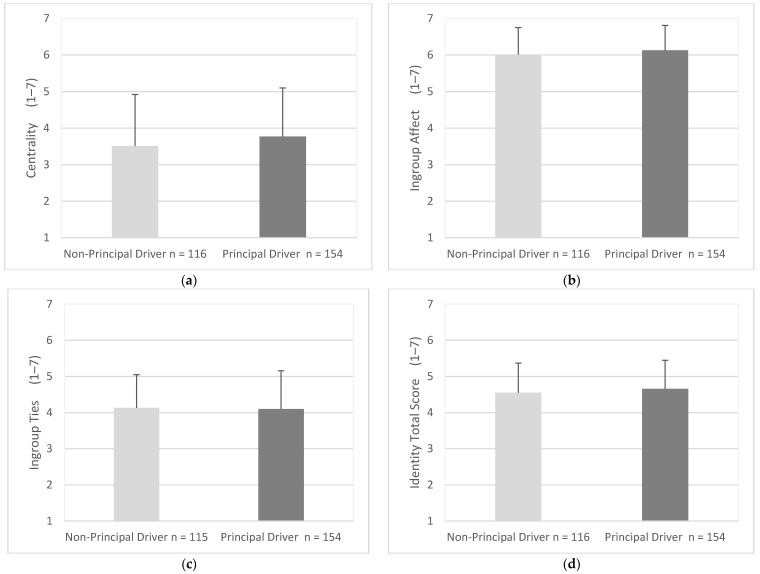
Mean score for identity factors and total scores as endorsed by principal and non-principal drivers. Items were measured using responses of 1 (strongly disagree) to 7 (strongly agree). Error bars represent standard deviation. (**a**) Centrality, (**b**) Ingroup Affect, (**c**) Ingroup Ties, (**d**) Identity Total Score.

Centrality scores were similar for principal drivers (M = 3.77, SD = 1.33) and non-principal drivers (M = 3.51, SD = 1.41), (95% CI, −0. 59 to 0.07), t (268) = 1.55 *p* = 0.122 (Figure 9a). Ingroup affect scores were similar for principal drivers (M = 6.13, SD = 0.68) and non-principal drivers (M = 6.01 SD = 0.74), (95% CI, −0.28 to 0.05), t (268) = 1.35, *p* = 0.180 (Figure 9b). Ingroup ties scores were similar between principal drivers (M = 4.10, SD = 1.06) and non-principal drivers (M = 4.13, SD = 0.92), (95% CI, −0.21 to 0.28), t (267) = 0.21, *p* = 0.783 (Figure 9c). Total scores for Identity were similar for principal drivers (M = 4.57, SD = 0.83) and non-principal drivers (M = 4.69, SD = 0.75), (95% CI, −0. 27 to 0.04), t (400) = 1.47, *p* = 0.239 (Figure 9d).

**Figure 10:**
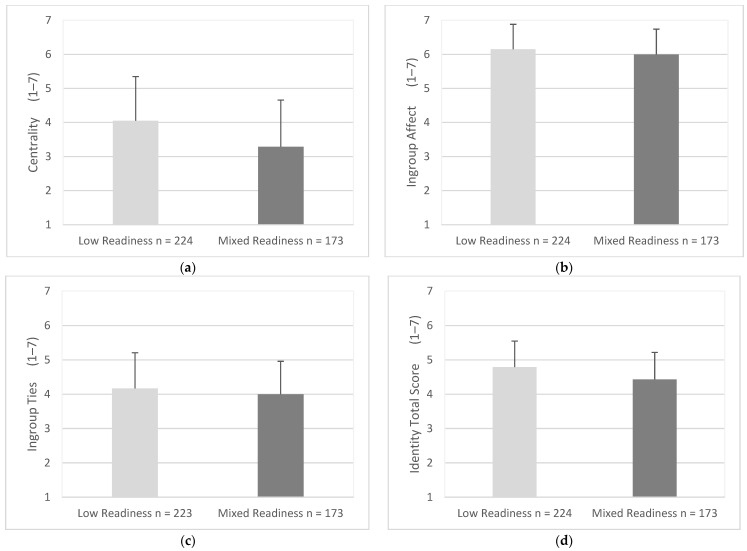
Mean score for identity factors and total scores as endorsed by participants for low readiness and mixed readiness for mobility change. Items were measured using responses of 1 (strongly disagree) to 7 (strongly agree). Error bars represent standard deviation. (**a**) Centrality, (**b**) Ingroup Affect, (**c**) Ingroup Ties, (**d**) Identity Total Score.

Centrality scores were statistically higher for drivers with low readiness scores (*M* = 4.05, *SD* = 1.30) when compared with drivers with mixed readiness scores (*M* = 3.29, *SD* = 1.37), (95% CI, −0. 50 to 1.03), *t* (395) = 5.65, *p* = 0.004 (Figure 10a). Ingroup affect scores were statistically higher for drivers with low readiness scores (*M* = 6.15, *SD* = 0.73) when compared with drivers with mixed readiness scores (*M* = 5.99, *SD* = 0.74), (95% CI, 0.01 to 0.30), *t* (395) = 2.13, *p* = 0.034 (Figure 10b). Ingroup ties scores were similar for drivers with low readiness scores and mixed readiness scores (*M* = 4.17, *SD* = 1.04) (*M* = 4.00, *SD* = 0.96), (95% CI, −0.37 to 0.36), *t* (394) = −1.61, *p* = 0.055 (Figure 10c). Total scores for identity for drivers with low readiness scores were statistically higher (*M* = 4.78, *SD* = 0.76) when compared with drivers with mixed readiness scores (*M* = 4.43, *SD* = 0.79), (95% CI, −0.21 to 0.51), *t* (395) = 4.58, *p* = 0.004 (Figure 10d).

### 3.8. Perception of Driving Cessation

**Figure 11:**
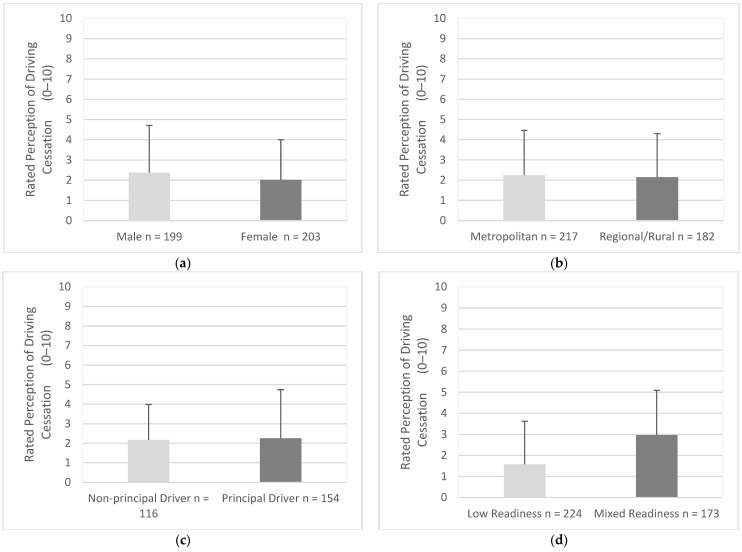
Perception of driving cessation as endorsed by participants in each group. Participants rated driving cessation with responses of 0 (extremely negative) to 10 (extremely positive). (**a**) Gender, (**b**) Residential Area, (**c**) Driving Roles, (**d**) Readiness.

The perception of driving cessation was generally negative for both male (M = 2.37, SD = 2.34) and female (M = 2.02 SD = 1.98), (95% CI, −0.08 to 0.77), t (400) = 1.60, *p* = 0.109 (Figure 11a). The perception of driving cessation was similarly negative for drivers in metropolitan areas (M = 2.25, SD = 2.21) and drivers in regional/rural areas (M = 2.15 SD = 2.15), (95% CI, −0.33 to 0.53), t (397) = 0.46, *p* = 0.647 (Figure 11b). The scores for the perception of driving cessation were generally negative for principal drivers (M = 2.17, SD = 1.82) and non-principal drivers (M = 2.26, SD = 2.48), (95% CI, −0.62 to 0.45), t (268) = −0.32, *p* = 0.749 (Figure 11c). Drivers with low readiness scores (M = 1.58, SD = 2.04), were significantly more likely to perceive driving cessation as more negative when compared to drivers with mixed readiness scores (M = 2.97, SD = 2.12), (95% CI, −1.80 to 0.98), t (395) = 6.63, *p* = < 0.001 (Figure 11d).

### 3.9. Resilience

**Figure 12:**
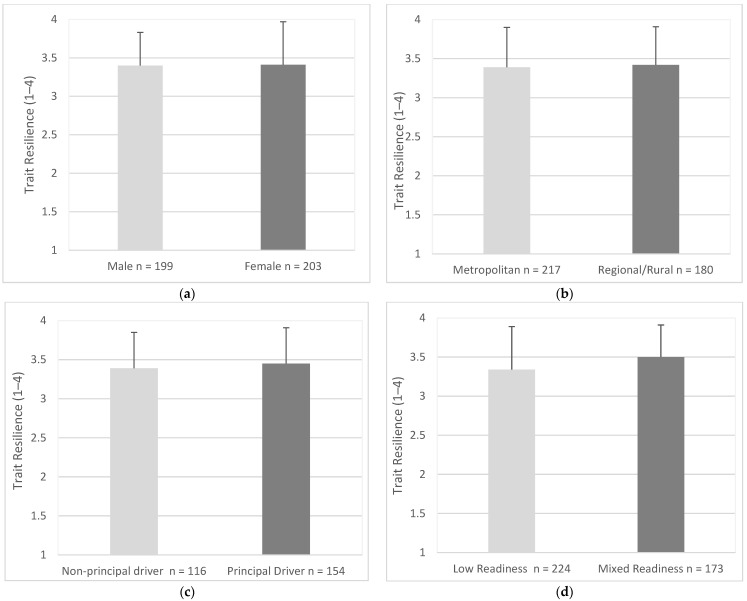
Trait Resilience as endorsed by participants in each group. Items were measured using responses of 1 (disagree) to 4 (strongly agree). (**a**) Gender, (**b**) Residential Area, (**c**) Driving Roles, (**d**) Readiness.

Trait resilience was similar for males (*M* = 3.40, *SD* = 0.43) and females (*M* = 3.41, *SD* = 0.56), (95% CI, −1.17 to 0.08), *t* (398) = 0.37, *p* = 0.714 (Figure 12a). Trait Resilience was similar for drivers in metropolitan areas (*M* = 3.39, *SD* = 0.51) and drivers in regional/rural areas (*M* = 3.42, *SD* = 0.49), (95% CI, −0.13 to 0.07), *t* (395) = 0.55, *p* = 0.585 (Figure 12b). Trait resilience was similar for principal drivers (*M* = 3.45, *SD* = 0.46) and non-principal drivers (*M* = 3.39, *SD* = 0.46), (95% CI, −0.62 to 0.45), *t* (268) = 0.32, *p* = 0.275 (Figure 12c). Trait resilience was significantly lower for drivers with low readiness scores (*M* = 3.34, *SD* = 0.55) when compared with drivers with mixed readiness scores (*M* = 3.50, *SD* = 0.41), (95% CI, −0.26 to −0.06), *t* (393) = 3.25, *p* = < 0.001 (Figure 12d).

### 3.10. Driver Identity and it’s Relationship with Driving Characteristics, Readiness for Mobility Change and Perception of Driving Cessation

Table 2 summaries the associations between each of the three identity factors and total score with driving characteristics, readiness perception of driving cessation, resilience.

Centrality scores were significantly and positively correlated with scores for ingroup affect *r_s_* (402) = 0.37, ingroup ties, *r_s_*(401) = 0.37, and identity total score, *r_s_* (402) = 0.84. Centrality scores were significantly and positively correlated with scores on the ARMT, *r*_s_(397) = 0.35 and driving frequency *r_s_* (401) = 0.12. Centrality scores were significantly and negatively correlated with perception of driving cessation (PDC), *r_s_* (402) = −0.20.

Ingroup affect scores were significantly and positively associated with ingroup ties, *r_s_* (401) = 0.31, and identity total score, *r*_s_(402) = 0.65. There were also significant positive correlations between ingroup affect scores and scores on the ARMT, *r_s_* (397) = 0.22, trait resilience, *r*_s_(400) = 0.27, driving frequency, *r*_s_(401) = 0.16 and driving standard, *r_s_* (401) = 0.21. There was also a negative correlation between ingroup affect scores and PDC, *r_s_* (402) = −0.29.

Ingroup ties scores were significantly and positively associated with identity total score, *r_s_* (401) = 0.71. There were also significant positive correlations between ingroup ties and, trait resilience, *r_s_* (399) = 0.15 and driving frequency, *r_s_* (400) = 0.12.

There were significant and positive correlations between identity total score and the ARMT, *r_s_* (397) = 0.29, trait resilience *r_s_* (400) = 0.10, accessibility to alternative transport *r_s_* (379) = 0.10, driving frequency, *r_s_* (401) = 0.17 and driving standard, *r_s_* (401) = 0.11.

Scores on the ARMT were significantly and negatively correlated with PDC, *r_s_* (397) = −0.47. There were also significant negative correlations between scores on the ARMT and, trait resilience *r_s_* (395) = −0.13, self- reported health, *r_s_* (397) = −0.13, whether someone minded doing the majority of driving, *r_s_* (151) = −0.21 and driving standard, *r_s_* (396) = −0.12.

## 4. Discussion

This study aimed to understand the psychosocial dynamics of driving. More specifically, we aimed to examine the differences in driving characteristics (i.e., history, roles: principal or non-principal driver, frequency, perception of driving standard, availability of alternative transport), driver identity, perception of driving cessation, resilience across gender, residential area, principal, and non-principal drivers and individuals with different degrees of readiness for mobility transition? Additionally, we aimed to examine characteristics associated with driver identity and whether driver identity impacted people’s self-reported readiness for mobility change and perception of driving cessation.

Driving history was comparable between residential areas, principal and non- principal drivers and drivers with low and mixed readiness for mobility change. However, there was a noticeable difference in driving history between genders, with males having more driving experience than females. Additionally, males self-reported most of the driving in households where other drivers were present and drove on a daily basis, more often than females. Males were also more likely to suggest their driving was better than other people of the same age and gender than females. Taken together, these findings seem to align with the pervasive cultural imagery of masculinity, driving and the need to be successful and in control [18].

A lack of alternative transport is often found in regional and rural communities [36,37]. However, in this sampled population alternative transport seemed to be limited across all groups including gender, residential area, principal or non-principal household drivers and people with low and mixed readiness for mobility change. In the past, a lack of viable alternative transport was associated with a lack of planning for driving cessation [38,39]. Indeed, the present findings seem to suggest that many people have concerns and or have engaged in little planning for driving retirement.

According to Meuser et al. [31], people with low readiness for mobility change are less likely to be prepared for driving retirement and are more likely to report feeling threatened by the prospect of driving cessation. This sample of individuals with low readiness for mobility change perceived driving cessation more negatively and self- reported lower trait resilience than those with mixed readiness for mobility change. Individuals with low readiness for mobility change also had higher scores of centrality, ingroup affect and total scores for identity. There were no significant differences between males and females across these identity scores. Further, males and females reported a similar level of readiness for mobility transition. These findings seem to suggest that people with more concerns for mobility transition may think about and have more of an emotional investment regarding driving.

Indeed, Musselwhite and Haddad [8] found that the motivations for travel in older adults were found to be related to three distinct needs: utilitarian needs, affective needs and aesthetic needs. Despite utilitarian needs often being discussed first, through subsequent interviews, participants seemed to become more aware of these other needs [8]. According to Musselwhite and Haddad [8], utilitarian needs seemed to be related to the practical aspects of driving, like accessibility and efficiency. Affective needs seemed to be related to psychosocial factors like self-agency, the fulfilment of social obligations, status and a sense of belonging. Aesthetic needs seemed to be related to the importance of making a journey for its own sake. Driving appeared to be an intrinsically pleasurable experience for some participants, often used for exploration or relaxation.

Driving cessation was generally perceived as a negative change across all groups. However, those who had low readiness for mobility change seemed to have felt more negatively towards driving cessation. Ethier and Deaux [40] have suggested that irrespective of whether a change was negative or positive, people will initially experience a feeling of instability because it can be a significant departure from what they are accustomed to. Similarly, people may initially perceive driving cessation as a negative change because it requires significant adjustments to their lifestyle. Moreover, driving cessation is often associated with the onset of older age and dependency characteristics that have negative implications in society [5,28,29]. For those with a low readiness for mobility change driving cessation was viewed more negatively comparatively to other groups. People with low readiness were characterized by higher scores of centrality and ingroup affect which could suggest greater emotional investment in the driving role. There were no significant differences for identity scores across this sample of older males and females. While principal and non-principal drivers did not differ in identity, principal drivers tended to be less ready for mobility transition compared to non-principal drivers, once again highlighting a degree of emotional investment involved in “driver” identity.

### Limitations

In this study the ARMT was used to evaluate a possible link between readiness for mobility change and identity however typically the measure is administered in clinical settings. In future studies it may be beneficial to adopt an interview like method as there may be more opportunities to establish rapport and elicit more intimate responses from participants.

With the spread of COVID-19, car use has been reduced across the world and this means people may be less able to fulfill family obligations as they normally would [41,42]. The present findings in this cross-sectional survey assessment must be considered in light of this context. Indeed, Abootalebi and colleagues [41] have suggested that while driving retirement is typically painful in and of itself, with the negative health messaging and stay-at-home restrictions, etc., due to the pandemic, the emotional discomforts surrounding driving cessation are likely to be exacerbated.

## 5. Conclusions

Driving is an important form of independence and autonomy, however driving cessation may represent more than loss of transportation, it can be threatening to an individual’s self-identity and self-perceptions of their own aging. Several of the findings reported here are novel. Low readiness for mobility change was linked with driving cessation being perceived as a more negative life event in this sample of older people. While there was a noticeable difference in driving history and frequency, with males reporting more experience and spending more time driving than females, there were no differences in driver identity measures between male and female participants. Further, both males and females perceived driving cessation as a negative event and reported similar levels of readiness for transition to non-driving.

These preliminary findings may aid in developing better contingency plans for those at risk of experiencing adverse outcomes following driving cessation. The findings suggest a need to provide appropriate resources to prompt conversations about and supports for individuals based on whether negative perceptions of driving cessation are perceived as a practical or emotional loss. For example, if people perceive driving as ‘a means to an end’, then a needs-based intervention, such as finding suitable alternative modes of transportation, may be suitable. If the transition involves an identity shift, then, without intervention, it could have negative impacts on an individual’s sense of self. In which case, intervening through supportive goal setting of personally relevant objectives and valued activities following driving cessation may be effective in lessening some of the negative identity-based effects of giving up driving.

Further work, exploring driver identity with groups of older adults with varying experiences, including those contemplating retiring from driving and recently retired drivers, is required. Co-designing studies and measures of driver identity with older people, will enhance further explorations.

## Figures and Tables

**Table 1:** Demographic Variables of the sample.

Variable		N (%)
Gender	Male	205 (50%)
	Female	205 (50%)
Employment	Employment full-time	94 (23%)
	Employment part-time	63 (15%)
	Employment casual/temporary (non-fixed hours)	17 (4%)
	Retired	213 (52%)
	Volunteer	2 (0.5%)
	Not currently employed/job seeking	21 (5%)
Education	Less than a high school diploma/certificate	0 (0%)
	High school diploma/certificate	63 (15%)
	Trade/Technical School	30 (7%)
	Associate degree (e.g., AA, AS)	50 (12%)
	Bachelor’s degree (e.g., BA, BS)	138 (34%)
	Master’s degree (e.g., MA, MS, Med)	92 (22%)
	Doctorate or professional degree (e.g., MD, DDS, PhD	30 (7%)
	Other	7 (2%)
Residential Area	Metropolitan	221 (54%)
	Regional/rural	185 (46%)
Income sufficiency	It is impossible	9 (2%)
	It is always difficult	51 (12%)
	It is sometimes difficult	105 (26%)
	It is not too bad	148 (36%)
	It is easy	97 (24%)
Health	Much worse	6 (2%)
	Worse	67 (16%)
	About the same	123 (30%)
	Better	149 (36%)
	Much better	65 (16%)
Driving as Primary Transport	No	24 (6%)
	Yes	386 (94%)
Access to alternative transport	No	275 (72%)
	Yes	110 (29%)
Other Drivers in household	No	133 (32%)
	Yes	277 (68%)
Principal Driver (if other drivers in household)	No	119 (43%)
	Yes	158 (57%)
Driving Frequency	Less than once per month	17 (4%)
	At least once a month	16 (4%)
	At least once a week	66 (16%)
	Several days per week	167 (41%)
	Daily	143 (35%)
Readiness	Low Readiness	224 (55%)
	Mixed Readiness	173 (43%)
	High Readiness	8 (2%) **

** People with high readiness (8) were not included in subsequent analyses due to sample size.

**Table 2:** Spearman correlations between driving characteristics, readiness for mobility change and perception of driving cessation.

	CEN	IGA	IGT	ID-TS	ARMT	PDC	RS	ACC	HLTH	DH	MND	FREQ	STAN
CEN		0.37 *	0.37 *	0.84 *	0.35 *	−20 *	−0.10	0.07	−0.07	0.04	−0.11	0.012	0.04
IGA			0.32 *	0.66 *	0.22 *	−0.30	0.27 *	0.08	0.10	0.07	0.30 *	0.16 *	0.21 *
IGT				0.71 *	0.08	−0.01	0.150 *	0.074	0.08	0.09	0.03	0.12 *	0.05
ID-TS					0.29 *	−0.20 *	0.10	0.10	0.03	0.09	0.05	0.17 *	0.011
ARMT						−0.47 *	−13 *	−0.06	−0.13 *	−0.04	−0.21 *	0.02	−0.12 *
PDC							−0.01	0.06	0.07	−0.03	0.07	−0.06	.
RS								0.08	0.42 *	0.07	0.27 *	0.18 *	0.31 *
ACC									0.18 *	0.057	−0.04	0.15 *	0.13 *
HLTH										0.17 *	0.22 *	0.24 *	0.27 *
DH											0.10	0.01	0.16 *
MND												0.07	0.19 *
FREQ													0.19
STAND													

*Note.* Non-parametric Spearman’s correlations. CEN = Centrality, IGA = Ingroup Affect. IGT = Ingroup Ties, ID-TS = Identity Total Score. ARMT = Assessment for Readiness for Mobility Transition Scale. PDC = Perception of Driving Cessation. RS = Resilience, Acc = Accessibility to Alternative Transport, Self-reported health = Hlth. DH = Driving History, MND = Mind doing the majority of driving in household. Freq = Frequency, Stand = Standard. * *p* < 0.05.

## Data Availability

Not applicable.
